# Periodontal treatment to improve glycaemic control in diabetic patients: study protocol of the randomized, controlled DIAPERIO trial

**DOI:** 10.1186/1745-6215-10-65

**Published:** 2009-08-02

**Authors:** Jean-Noel Vergnes, Elise Arrivé, Pierre Gourdy, Hélène Hanaire, Vincent Rigalleau, Henri Gin, Cyril Sédarat, Georges Dorignac, Christophe Bou, Michel Sixou, Cathy Nabet

**Affiliations:** 1 Department of Dentistry, Toulouse University Hospital (CHU de Toulouse) and Toulouse Dental School, Paul Sabatier University, Toulouse, France; 2 Department of Dentistry and Oral health, Bordeaux Teaching Hospital - Department of Odontology, Bordeaux 2 University, France; 3 Diabetology Department, Cardiovascular and Metabolic Unit, Rangueil University Hospital, Toulouse, France; 4 Department of Nutrition and Diabetology, Bordeaux Teaching Hospital, Bordeaux 2 University, France

## Abstract

**Background:**

Periodontitis is a common, chronic inflammatory disease caused by gram-negative bacteria leading to destruction of tissues supporting the teeth. Epidemiological studies have consistently shown increased frequency, extent and severity of periodontitis among diabetic adults. More recently, some controlled clinical trials have also suggested that periodontal treatment could improve glycaemic control in diabetic patients. However current evidence does not provide sufficient information on which to confidently base any clinical recommendations. The main objective of this clinical trial is to assess whether periodontal treatment could lead to a decrease in glycated haemoglobin levels in metabolically unbalanced diabetic patients suffering from chronic periodontitis.

**Methods:**

The DIAPERIO trial is an open-label, 13-week follow-up, randomized, controlled trial. The total target sample size is planned at 150 participants, with a balanced (1:1) treatment allocation (immediate treatment vs delayed treatment). Periodontal treatment will include full mouth non-surgical scaling and root planing, systemic antibiotherapy, local antiseptics (chlorhexidine 0.12%) and oral health instructions. The primary outcome will be the difference in change of HbA1c between the two groups after the 13-weeks' follow-up. Secondary outcomes will be the difference in change of fructosamine levels and quality of life between the two groups.

**Discussion:**

The DIAPERIO trial will provide insight into the question of whether periodontal treatment could lead to an improvement in glycaemic control in metabolically unbalanced diabetic patients suffering from periodontitis. The results of this trial will help to provide evidence-based recommendations for clinicians and a draft framework for designing national health policies.

**Trial registration:**

Current Controlled Trials ISRCTN15334496

## Background

Periodontitis is a common, chronic inflammatory disease caused by gram-negative infection leading to the destruction of tissues supporting the teeth. Approximately 50% of adults in the United States have chronic periodontitis [1] and similar rates have been reported in France [2].

Diabetes is a metabolic disorder characterized by high blood glucose levels. It is a significant cause of morbidity and mortality in both developed and developing countries. Its prevalence for all age-groups worldwide has been estimated at 2.8% in 2000 and 4.4% in 2030 [3].

The relationship between these two affections has been largely described in the literature over the past 50 years. Epidemiological studies have consistently shown increased frequency, extent and severity of periodontitis among diabetic adults [4]. More recently, some controlled clinical trials have also suggested that periodontal treatment could improve glycaemic control in diabetic patients [5-10]. We previously conducted a systematic review of the literature and found that results were conflicting across trials [11].

This could be attributed to considerable differences in methodology and also in the sample sizes and compositions of the groups included in the studies. The study by Jones et al. [6] was the best quality study that we assessed in the meta-analysis but the subjects enrolled in this trial were highly specific (American veterans, mainly males, smokers, with poorly controlled diabetes). Other studies showed evidence of methodological deficiencies (mainly small sample sizes). Further, the majority of clinical trials on this topic have included subjects with poorly controlled diabetes (HbA1c > 9%) [11]. We argue that periodontal treatment to improve glycaemic control in diabetic patients would be more relevant among patients with moderately poor glycaemic control (HbA1c between 7.0 and 9.5%). Interventions other than periodontal treatment (e.g. adequate medical treatment) are likely to be more effective in reducing HbA1c levels in poorly controlled diabetic patients.

Our systematic review emphasized the need to conduct a well-designed randomized controlled trial in a European population, among patients with suboptimal glycated haemoglobin levels, according to clinical recommendations for the treatment of chronic periodontitis in diabetic subjects.

## Methods

The DIAPERIO trial is an open-label, 13-week follow-up, randomized, controlled trial. The design specifies a balanced (1:1) treatment allocation.

### Objectives

The main objective of this study is to assess whether periodontal treatment could lead to a decrease in glycated haemoglobin (HbA1c) levels in metabolically unbalanced diabetic patients suffering from periodontitis. We will therefore test the null hypothesis that periodontal treatment does not reduce the HbA1c level in metabolically unbalanced diabetic patients suffering from periodontitis.

The secondary objectives of this study are to assess, among metabolically unbalanced diabetic patients suffering from periodontitis:

• whether periodontal treatment could lead to a decrease in fructosamine levels,

• whether periodontal treatment could lead to an improvement in quality of life.

### Study participants

#### Setting and location

**Figure 1:**
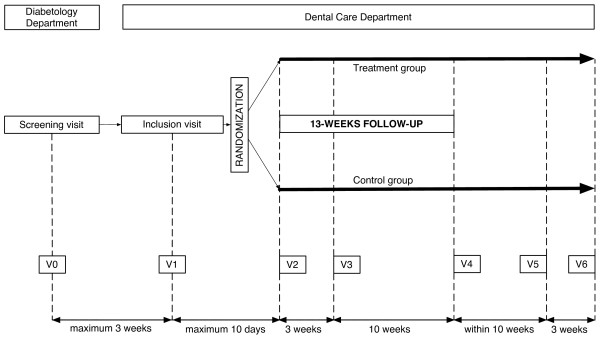
**Study timelines**.

Volunteers will be recruited in the Diabetology Departments of two University Hospitals of south-western France (Toulouse-Rangueil Teaching Hospital and Bordeaux-Haut-l'Eveque Teaching Hospital). Subjects will be selected in two steps: a screening visit at the Diabetology Department, and an inclusion visit at the Dental Care Department of the same University Hospital. Periodontal treatments and follow-up visits will be performed in the Dental Care Departments (Figure 1).

#### Eligibility criteria

Eligibility criteria will be assessed in the Diabetology and Dental Care departments at V0 and V1 respectively.

##### At V0 (Diabetology Department)

During the study period, all consecutive patients who consult in the Diabetology Departments will be screened for possible inclusion in the trial. Pre-inclusion and non-inclusion criteria will be checked by diabetologists using a standardized questionnaire.

##### Pre-inclusion criteria (V0)

To be pre-included, patients will have to:

- be aged 18 years or older (male or female),

- be affiliated to a public health system,

- be diagnosed as having had type 1 or 2 diabetes for at least one year before V0,

- have a last known value of HbA1c, measured within three months prior V0, between 6.8% and 9.7%,

- have received stable antidiabetic therapy (no changes to diet, medication, dosage or formulation) during the three months preceding V0,

- have at least six natural permanent teeth,

- be available for all study visits over three months in the Dental Care Departments (V1 to V6),

- be able to give their written informed consent.

##### Non-inclusion criteria (V0)

Several non-inclusion criteria will also be checked in the Diabetology Departments. Patients will not qualify for enrolment if they:

- have severe difficulties in understanding written and spoken French,

- are participating in another intervention study,

- do not have a known value of HbA1c level,

- have a known diabetes complication leading to planned hospitalization within 4 months after the screening visit,

- have a known pathological condition leading to planned hospitalization within 4 months after the screening visit,

- are suffering from one or more known infectious diseases (HIV, hepatitis, infectious mononucleosis),

- are suffering from known clinically significant renal disease (creatinine clearance <60 ml/min), or liver disease,

- have known phenylketonuria,

- have a known risk of endocarditis,

- have a permanent pacemaker,

- suffer from a chronic disorder that requires chronic or intermittent use of corticosteroids and/or antibiotics,

- are taking antithrombotic treatment,

- have known hypersensitivity to chlorhexidine gluconate,

- have known contraindications to both amoxicillin and clindamycin,

- for females: are pregnant or intending to become pregnant, or lactating.

Subjects satisfying the pre-inclusion criteria and exempt from the non-inclusion criteria at V0 will be given the possibility of further participating in the trial and will be given a scheduled appointment at the Dental Care Department (V1) within 3 weeks after V0 (Figure 1).

##### At V1 (Dental Care Department)

Non-inclusion and inclusion criteria will be checked by trained dentists-examiners, using a standardized questionnaire and a calibrated examination method.

##### Non-inclusion criteria (V1)

At this stage, patients will not qualify for enrolment if they present at least one of the following: acute oral infection, acute oral pain (including pulpitis), suspicious oral mucosal lesion, severe oral inflammation unrelated to periodontal conditions, or need for immediate tooth extractions. Patients with any of the above problems will be offered treatment in the Dental Care Department as routine patients. Inclusion will be reconsidered after remission of the condition(s).

##### Inclusion criteria (V1)

Final inclusion will be decided at the Dental Care Departments, during the inclusion visit (V1). The criteria for inclusion are:

- written informed consent,

- no change relative to pre-inclusion criteria and non-inclusion criteria since V0,

- patient with periodontitis. Periodontitis is defined by the presence of one site with periodontal probing depth (PPD) ≥ 4 millimetres and clinical attachment level (CAL) ≥ 3 millimetres on at least 4 teeth.

- patient with HbA1c level ≥ 7.0% and ≤ 9.5%. Blood samples will be taken during V1, after periodontal assessment. Thus blood will only be taken from patients with periodontitis.

Subjects fulfilling all the inclusion criteria and none of the non-inclusion criteria at V1 will be randomized and will be given a scheduled appointment at the Dental Care Department, within 10 days for the treatment group (V2), and between 21 and 31 days from V1 for the control group (V3) (Figure 1).

### Intervention

Periodontal treatment will include non-surgical scaling and root planing, systemic antibiotherapy, and oral health instructions.

#### • Scaling and root planing (SRP)

This will be performed at V2 for the treatment group, and at V5 for the control group (Figure 1). Non-surgical periodontal therapy will be performed during a 2-hour, single-session, full-mouth ultrasonic debridement by trained dentists-examiners, who will receive special training from a periodontist prior to the beginning of the study.

Disclosing solution will be used to visualize the plaque for the clinician. Then scaling and root planing will involve removal of supra- and sub-gingival plaque from all tooth surfaces, and removal of supra- and sub-gingival calculus with both ultrasonic and hand instruments (Gracey curettes). Mechanical debridement will be performed under local anaesthesia if necessary. Scaling and root planing will be combined with subgingival irrigation using an antiseptic mouth rinse (chlorhexidine 0.12%). Finally, the coronal and radicular surfaces of the teeth will be polished.

#### • Systemic antibiotherapy (ATB)

The French health authorities recommend the prescription of systemic antibiotherapy for metabolically unbalanced diabetic patients receiving periodontal treatment [12,13]. On primary intention, capsules of 500 mg amoxicillin will be prescribed to patients 3 times a day (morning, midday and evening) for 7 days. Two boxes of 12 capsules will be given to the patients after scaling and root planing (at V2 for the treatment group and at V5 for the control group). In case of contraindication to beta-lactam antibiotics, capsules of 300 mg clindamycin will be prescribed to patients twice a day (morning and evening) for 7 days. One box of 16 capsules will be given to the patients after scaling and root planing (at V2 for the treatment group and at V5 for the control group). These antibiotics are recommended by French health authorities for the treatment of chronic periodontitis in diabetic patients [13].

#### • Oral health instructions (OHI)

After periodontal treatment, subjects will receive oral health instructions from the dentist that performed the scaling and root planing. These instructions will be given at V2 and V3 for the treatment group and at V5 and V6 for the control group. A 15-minute oral session will include verbal and visual information on how to use toothbrush, floss and interspace brushes, how to clean bridges and dentures, how and why to use a plaque disclosing test and a mouthwash.

A pack containing all the dental care products necessary for 3-months of periodontal maintenance will be given to the subjects at V2 for the treatment group and V5 for the control group. It will include three 75 ml tubes of toothpaste, three soft toothbrushes, 12 single-tufted interspace brushes, and sufficient dental floss and plaque disclosing tablets for a daily use over 3 months. An antiseptic mouthwash (chlorhexidine 0.12%) will also be given for mouth washing three times a day for 10 days after the scaling and root planing session.

Finally, the pack will contain leaflets that will provide pictorial information relative to the method, the frequency and the duration of each procedure.

Subjects in the control group will receive neither periodontal treatment nor a placebo during the 3-months follow-up (from V1 to V4). They will receive the same periodontal treatment as subjects in the treatment group (non-surgical scaling and root planing, systemic antibiotherapy, two-sessions of oral health instructions and antiseptic prescription) at V5 and V6 (Figure 1).

### Outcomes

#### HbA1c

The primary outcome of the DIAPERIO trial will be the difference in change of HbA1c from V1 to V4 between the two groups. The HbA1c level is proportional to average blood glucose concentration over the previous 1 to 3 months.

#### Fructosamine

A secondary outcome will be the difference in change of fructosamine levels from V1 to V3 and from V1 to V4 between the two groups. Fructosamine reflects an average of blood glucose levels over a period of 2 to 3 weeks.

#### Quality of life

A pragmatic outcome will be the difference in change in the quality of life between the two groups from V1 to V4. Quality of life will be assessed using the SF-36 questionnaire.

### Study assessments

#### Periodontal status

A full-mouth periodontal examination will be performed by trained dentists-examiners at V1 and V4 for both groups. The clinical examination will include depth of periodontal pockets (PPD in mm), level of clinical attachment (CAL in mm) and bleeding on probing (BOP: yes/no). Values will be taken for all scorable teeth at six sites per tooth (mesio-buccal, mid-buccal, disto-buccal, mesio-lingual, mid-lingual and disto-lingual). No radiographs will be taken. The result of the periodontal treatment will be assessed on the healing response in the tissues using PPD, CAL, BOP, and the amount of inflamed periodontal tissue (PISA)[14].

#### Questionnaires

At V1 and at V4, subjects will complete a questionnaire designed to gauge medical, social and oral health behavioral information. The questionnaire completed at V4 will assess the evolution of oral health behavior during the 3-months follow-up. Adverse events will also be reported. Reportable adverse events will include unexpected study-related events (oral adverse events) and serious adverse events regardless of cause.

### Randomization

Randomization will be blocked by centre. Subjects meeting eligibility criteria at V1 will be randomly assigned to receive periodontal treatment within 10 days (treatment group) or delayed periodontal treatment (control group) with the use of a block stratified according to clinical centre.

### Study timelines

The DIAPERIO trial will include 4 phases: pre-inclusion, inclusion-randomization, follow-up and post-follow-up (Figure 1). Maximum study length per participant will be 30 weeks. No longer term follow-up is planned. Table 1 shows the study schedule. V2 only concerns subjects from the treatment group, while V5 and V6 only concern subjects from the control group (delayed treatment).

**Table 1:** Study schedule.

	V0	V1	V2	V3	V4	V5	V6
**Treatment group**							

Review of pre-inclusion criteria	*						

Review of inclusion criteria		*					

Written informed consent		*					

Full periodontal assessment		*			*		

Initial questionnaire + SF-36		*					

Blood collection (HbA1c)		*			*		

Blood collection (fructosamine)		*		*	*		

Periodontal treatment (SRP)			*				

Oral hygiene instructions			*	*			

Final questionnaire + SF-36					*		

Appointment for a next visit	*	*	*	*			

**Control group**							

Review of pre-inclusion criteria	*						

Review of inclusion criteria		*					

Written informed consent		*					

Full periodontal assessment		*			*		

Initial questionnaire + SF-36		*					

Blood collection (HbA1c)		*			*		

Blood collection (fructosamine)		*		*	*		

Periodontal treatment (SRP)						*	

Oral hygiene instructions						*	*

Final questionnaire + SF-36					*		

Appointment for a next visit	*	*		*	*	*	

### Statistical methods

Sample size calculation was based on detecting a difference of 0.5% in HbA1c change from baseline between the two groups. Assuming a standard deviation of 1.0% and using a 2-sided test at 5% significance level, 64 participants per group would yield 80% power. Anticipating a 15% dropout rate, the target sample size was thus planned at 150 participants.

Baseline characteristics by group will be presented as mean (SD) for continuous variables and tallied for categorical variables. The intention-to-treat population will consist of all randomized subjects whereas the evaluable population will consist of subjects from the intention-to-treat population who complete the 3-months follow-up. Descriptive statistics will be provided for the intention-to-treat population.

All primary analyses will be performed according to intention-to-treat principles, on the evaluable population (i.e. all patients with initial (V1) and final (V4) HbA1c measurements), regardless of the subject's presence at intermediate visits or his/her degree of compliance with the treatment. Subjects with missing initial or final HbA1c measurements will be excluded from the analysis. The main analysis will be a comparison between the two study groups concerning the change in the HbA1c levels from baseline to 3 months in an analysis of covariance (ANCOVA) model, adjusted for the baseline HbA1c level and clinical centre. Residual values will be examined for an approximate normal distribution. If HbA1c values are highly skewed, a transformation or non-parametric method will be used instead [15].

The data will also be analysed using the "per protocol" approach. Here the analysis will be restricted to participants who were compliant with the treatment protocol (SRP, ATB and OHI).

In addition to the primary analysis, five binary outcomes for HbA1c at 3 months (a relative decrease of ≥10%, a three-month level of < 7%, an absolute decrease of ≥0.5%, a relative increase of ≥10% and an absolute increase of ≥0.5%) will be evaluated in logistic regression models adjusted for the baseline HbA1c level and clinical centre [16].

For secondary outcomes, all continuous variables will be analysed via analysis of covariance using a model similar to that specified for the primary outcome. Analyses of binary variables will be based on logistic regression models adjusted for group and clinical centre. For secondary analyses, missing values will be imputed using the Last Observation Carried Forward method. The safety and efficacy of periodontal treatment will be assessed [17], as will the correlation between clinical response to periodontal treatment and HbA1c levels [18]. The proportion of subjects who had one or more adverse events in the two groups will be compared by means of Fisher's exact test.

### Ethical aspects

All participants will have to provide written informed consent. The informed consent form will contain the following information: names and affiliations of investigators, a plain language description of the study (treatment group, control group and intervention), the duration of the study, the right to withdraw at any time, the ethics committee approval and the privacy guarantee. All participants will receive free periodontal treatment (immediate or delayed).

The protocol and procedures have been approved by ethics and regulatory agencies and are implemented in accordance with provisions of the Declaration of Helsinki and French Good Clinical Practice guidelines for periodontal treatment in diabetic patients. The appropriate Committee for Protection of Research Subjects (Comité de Protection des Personnes [CPP], Sud-Ouest Outre-Mer I), approved the protocol on February 23rd, 2009. The Sanitary Safety of Health Products Agency (Agence française de sécurité sanitaire des produits de santé [AFSSAPS]), approved it on February 12th, 2009 (ref: 2008-A01467-48). The Advisory Committee for Data Processing in Health Research (Comité consultatif sur le traitement de l'information en matière de recherche dans le domaine de la santé [CCTIRS]), gave its approval on January 15th, 2009.

## Discussion

We aim to use a two-centre, randomized, controlled trial to investigate whether periodontal treatment could lead to an improvement in glycaemic control in metabolically unbalanced diabetic patients suffering from periodontitis. The results of this trial will help to provide evidence-based recommendations for clinicians and a draft framework for designing local and national health policies.

## Abbreviations

ATB: systemic antibiotherapy; BOP: bleeding on probing; CAL: clinical attachment level; HbA1c: glycated hemoglobin; OHI: oral health instructions; PISA: periodontal inflamed surface area; PPD: periodontal pocket depth; SRP: scaling and root planing.

## Competing interests

The authors declare that they have no competing interests.

## Authors' contributions

MS is the trial co-ordinator. JNV made substantial contributions to the conception and design of the study, and is co-responsible for the overall direction of the project, the analysis and interpretation of data. CN is co-responsible for the overall design, administration and direction of the study. PG and HH also participated in the design and direction of the study, and PG is the supervisor of the diabetology part of the research project. EA participated in the design of the study and is co-responsible with CB and GD for the coordination of the study in Bordeaux. CS is the supervisor of the periodontal part of the trial. VR and HG are supervisors of the diabetology part of the trial in Bordeaux. All authors have read and approved the final version.
